# Directional cell movements downstream of Gbx2 and Otx2 control the assembly of sensory placodes

**DOI:** 10.1242/bio.020966

**Published:** 2016-09-22

**Authors:** Ben Steventon, Roberto Mayor, Andrea Streit

**Affiliations:** 1 Department of Craniofacial Development, King's College London, Guy's Campus, Tower Wing Floor 27, London SE1 9RT, UK; 2 Department of Cell and Developmental Biology, University College London, Gower Street, London WC1E 6BT, UK

**Keywords:** Cell migration, Lens, Live imaging, Morphogenesis, Otic, Peripheral nervous system

## Abstract

Cranial placodes contribute to sensory structures including the inner ear, the lens and olfactory epithelium and the neurons of the cranial sensory ganglia. At neurula stages, placode precursors are interspersed in the ectoderm surrounding the anterior neural plate before segregating into distinct placodes by as yet unknown mechanisms. Here, we perform live imaging to follow placode progenitors as they aggregate to form the lens and otic placodes. We find that while placode progenitors move with the same speed as their non-placodal neighbours, they exhibit increased persistence and directionality and these properties are required to assemble morphological placodes. Furthermore, we demonstrate that these factors are components of the transcriptional networks that coordinate placode cell behaviour including their directional movements. Together with previous work, our results support a dual role for Otx and Gbx transcription factors in both the early patterning of the neural plate border and the later segregation of its derivatives into distinct placodes.

## Abstract (precis)

**Summary:** Using spatial and temporally controlled perturbations followed by live cell tracking *in vivo*, this paper demonstrates that directional movements downstream of Gbx2 and Otx2 are important for otic and lens placode formation.

## INTRODUCTION

Vertebrate cranial placodes give rise to crucial parts of the sensory nervous system including the olfactory epithelium, the inner ear and the sensory neurons of the cranial ganglia, as well as the lens (Schlosser, 2006; Streit, 2008). Initially, placode precursors occupy a unique territory, the pre-placodal region (PPR), where cells of different fates are interspersed (Bhattacharyya et al., 2004; Kozlowski et al., 1997; McCarroll et al., 2012; Streit, 2002; Xu et al., 2008; Pieper et al., 2011). Although it is controversial whether cell sorting segregates placode progenitors (Pieper et al., 2011), at later stages placodal cells must somehow coalesce to form spatially distinct placodes along the anterior-posterior axis (Breau and Schneider-Maunoury, 2014). In chick, DiI labelling reveals some movement of cell groups during otic, olfactory and lens placode formation (Bhattacharyya et al., 2004; Streit, 2002); while in zebrafish, cells move directionally in an integrin-α5 dependent manner as they are recruited into the otic placode (Bhat and Riley, 2011). Likewise, *Xenopus laevis* pre-placodal cells in the epibranchial region move directionally in response to the migration of adjacent neural crest cells (Theveneau et al., 2013). Whether or not these observed movements are a passive response to the morphogenesis of surrounding tissues, or directional movement as a consequence of cellular activities within the ectoderm itself, remains to be determined.

The transcription factor Gbx2 is required for otic specification, whereas Otx2 is needed for trigeminal, lens and olfactory specification (Steventon et al., 2012). Since both genes continue to be expressed as placodes are assembled they may mediate the coalescence of placode precursors (Hidalgo-Sánchez et al., 2000; Ogino et al., 2007; Tour et al., 2001). Therefore, we sought to repress Gbx2 and Otx2 targets in a spatially and temporally controlled manner to assess their role in the formation of otic and lens placodes. Using *Xenopus*, we show that whilst all cells within the deep ectoderm move at a similar velocity, placodal cells migrate with increased persistence to coalesce into distinct placodes, which in turn depends on Gbx2 and Otx2 downstream targets.

## RESULTS AND DISCUSSION

### Time-lapse imaging reveals the gradual emergence of sensory placodes

**Fig. 1.**
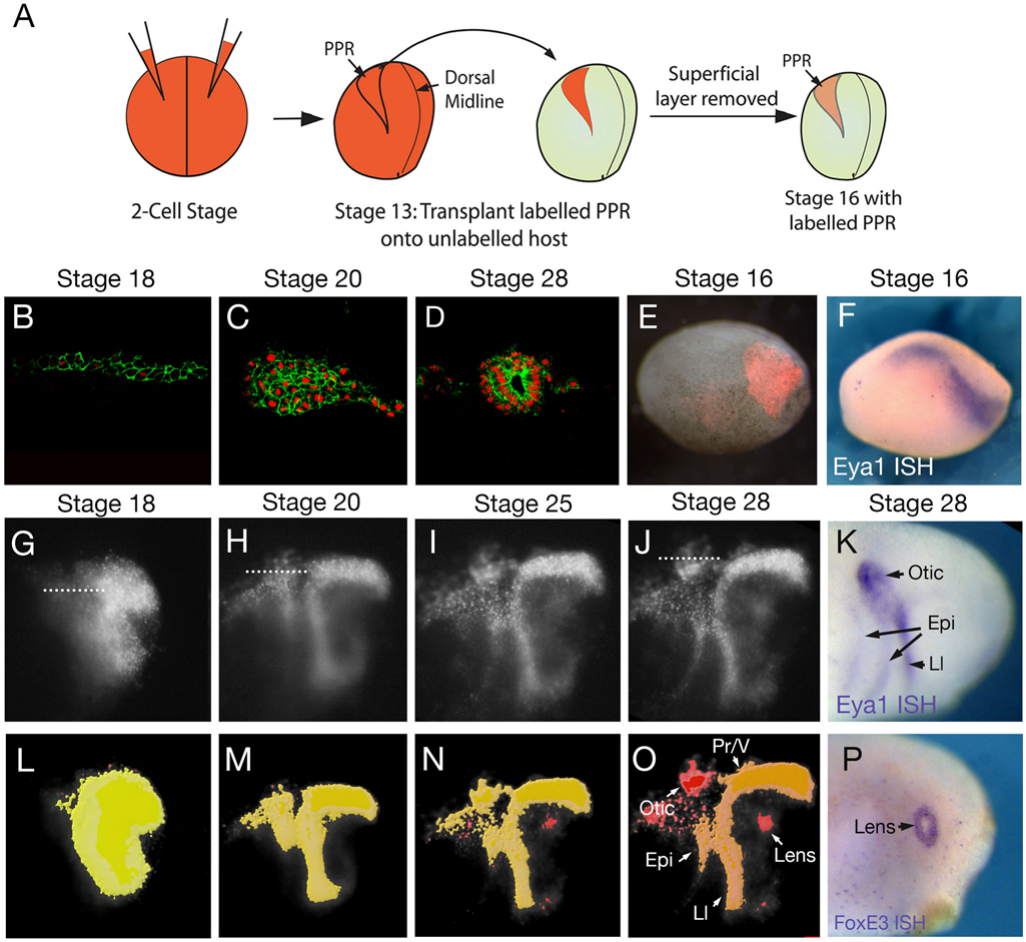
**Time-lapse imaging reveals the gradual emergence of sensory placodes.** (A) A schematic summarising the grafting technique. (B-D) Sections of PPR grafts injected with nRFP and mGFP in the otic region at the level indicated in G,H and J respectively. Cell outlines and nuclei are clearly visible, allowing visualisation of placode assembly. (E) Overlay of fluorescence and bright field image to reveal grafted cells at stage 16. (F) *Eya1* expression at stage 16. (G-J) Lateral images of nRFP labelled grafts between stages 19 and 28. Dotted lines in G,H,J indicate positions of sections shown in B-D. (K) *Eya1* expression at stage 28. (L-O) Automatically segmented regions of coherent cell labelling, colour-coded according to surface area, with a shift from yellow to red as the area decreases. Gradual segregation of placodal cells was observed in 8/8 grafted embryos. Arrows in (O) highlight individual placodes. (P) Expression of *FoxE3* at stage 28. Epi, epibranchial; Ll, lateral line; Pr/V, Profundal/trigeminal placodes.

Within the PPR of *Xenopus laevis*, the deep layer of the embryonic ectoderm contributes to the sensory placodes, while the superficial layer generates an epithelium that protects the embryo from the external environment (Chalmers et al., 2002). To visualize placode cell movements, we used a grafting approach to label the deep ectoderm specifically (Fig. 1A). Donor embryos were injected with mRNA encoding nuclear RFP (nRFP) alone or together with membrane GFP (mGFP) into both blastomeres at the two-cell stage. At stage 13, a region slightly larger than the PPR (compare grafted region in Fig. 1E with the expression of the PPR marker *Eya1* in a stage-matched embryo in Fig. 1F) was grafted into the same position of an unlabelled stage 13 host. At stage 16, the labelled superficial layer was removed and the un-labelled superficial ectoderm was allowed to heal (Fig. 1A). Sagittal sections through the otic region of embryos grafted with nRFP/mGFP injected PPR (at the levels indicated in Fig. 1G,H,J) show how the 2-3-cell deep ectoderm at the 18-somite stage (Fig. 1B) aggregates into a multi-layered cluster (Fig. 1C; 20-somite stage), before forming the otic vesicle by stage 28 (Fig. 1D). We performed time-lapse microscopy of embryos grafted with nRFP labelled PPR and segmented regions of coherent cells using the surface function of the image analysis tool Imaris (Fig. 1L-O; Movie 2). This analysis reveals a progressive subdivision from a homogeneous sheet of cells into regions of clustered nuclei that have a brighter fluorescence signal than the surrounding cells (Fig. 1G-O; Movie 1). As shown in Fig. 1B-D, placodes form as multi-layered aggregates of cells that are surrounded by a thin layer of non-placodal cells, therefore showing a brighter level of fluorescence when viewed in the whole embryo (Fig. 1G-J). These cell clusters match the shape and position of placodes closely, as assessed by the expression the posterior placode marker *Eya1* and the lens marker *FoxE3* in stage-matched embryos (Fig. 1K,P). The profundal/trigeminal and lateral line cells occupy a coherent domain that cannot be visually separated from surrounding non-placodal cells (Fig. 1O).

### Gbx2 and Otx2 targets are required for otic and lens placode assembly

**Fig. 2.**
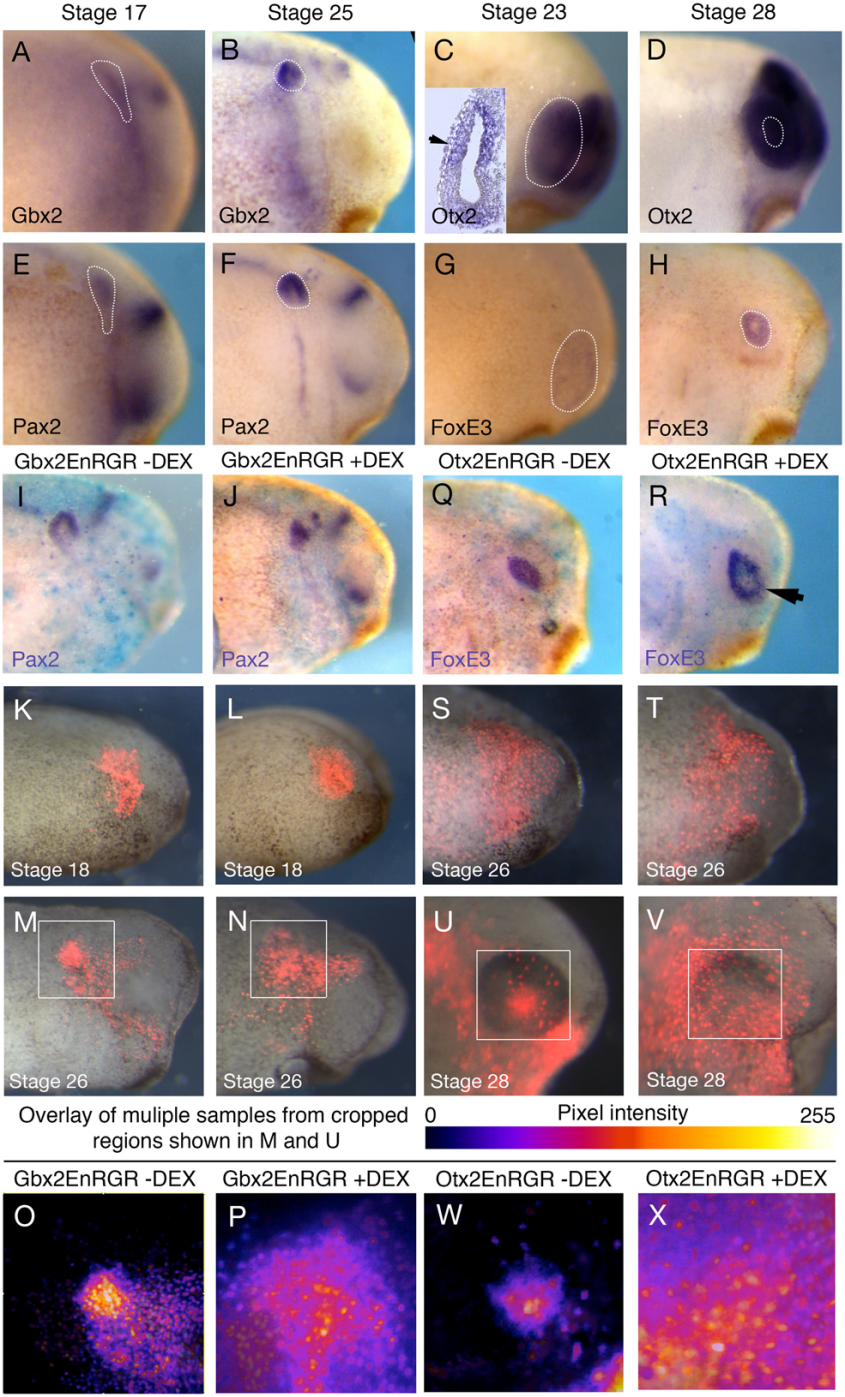
**Gbx2 and Otx2 are required for the correct assembly of otic and lens placodes.** (A-H) Expression of *Pax2* (A,B), *Gbx2* (C,D), *Foxe3* (E,F) and *Otx2* (G,H) at the stages indicated at the top of the panels. (I,J) *Pax2* expression in embryos with PPR grafts injected with Gbx2-EnR-GR mRNA in the absence (I) and presence of DEX (J). (K-N) Embryos with PPR grafts expressing Gbx2-EnR-GR and nuclear RFP at stage 18 (K,L) and cultured until stage 26 (M,N) embryos in the absence (K,M) or presence of DEX (L,N). (O,P) Intensity mapped overlays of 8 grafted embryos in the absence (O) and presence (P) of DEX. (Q,R) Embryos with PPR grafts injected with Otx2-EnR-GR mRNA in the absence (Q) and presence of DEX (R). (S-V) Embryos with PPR grafts expressing Otx2-EnR-GR and nuclear RFP at stage 26 (S,T) and cultured until stage 28 (U,V) in the absence (S,U) or presence of DEX (T,V). (W,X) Intensity mapped overlays of 8 grafted embryos in both the absence (W) and presence (X) of DEX.

The convergence of placode progenitors into morphological placodes as observed by our time-lapse analysis is mirrored by changes in *Gbx2* and *Otx2* expression: they are broadly expressed at neurula stages (Schlosser and Ahrens, 2004; Steventon et al., 2012) and continue to be expressed in the otic and lens placode at later stages (Fig. 2A-D) (Hidalgo-Sánchez et al., 2000; Ogino et al., 2007; Tour et al., 2001). The latter coincides with the expression of placode-specific genes like *Pax2* (Fig. 2E,F) in the otic domain and *Foxe3* in the lens (Fig. 2G,H). We therefore asked whether Gbx2 and Otx2 mediate placode assembly.

To manipulate Otx2 and Gbx2 function in a temporally controlled manner, we made use of hormone-inducible constructs where their homeodomain is fused to the engrailed repressor domain (EnR; Glavic, 2002). These constructs have previously been shown to mimic the effects of full length Otx2 and Gbx2 mRNAs in mid/hindbrain organiser positioning (Glavic et al., 2002) and in the subdivision of the pre-placodal region (Steventon et al., 2012). In addition, the Gbx2-EnR-GR construct rescues knock-down phenotypes of Gbx2 morpholinos (Li et al., 2009). Activation with dexamethasone (DEX) leads to the translocation of constitutive repressor forms into the nucleus thus causing repression of all or a sub-set of Otx2/Gbx2 target genes. To target the otic region, Gbx2-EnR-GR was injected into the A3 blastomere at the 32-cell stage. In the absence of DEX, expression of the otic marker *Pax2* at stage 26 is normal (Fig. 2I). In contrast, upon addition of DEX at stage 18, *Pax2* continues to be expressed, but the otic vesicle is of abnormal morphology (Fig. 2J). We next assessed cell behaviour using the same grafting strategy described above. Gbx2-EnR-GR mRNA was co-injected with nuclear RFP and the labelled posterior PPR was grafted into unlabelled hosts. In the absence of DEX, cells clearly aggregate in the otic region at stage 26 (Fig. 2K,M,O). However, upon addition of DEX, grafted cells remain spread out and are not incorporated into a placode structure (Fig. 2L,M,P). To combine data from multiple specimens, we overlaid images of the otic region from eight grafted control or experimental embryos (Fig. 2O,P) and displayed the results in a heat map summing the pixel intensity of all nRFP^+^-grafted cells. This shows that when Gbx2 target genes are repressed, cells remain distributed over a large area. However, this inhibition of otic placode formation is not complete because some cells do express *Pax2* within the normal otic placode region, albeit it with an abnormal morphology (Fig. 2J).

We performed similar experiments to repress Otx2 targets by injecting Otx2-EnR-GR into the A1 blastomere at the 32-cell stage to target the anterior PPR. As the lens placode forms at slightly later stages than the otic placode, and Otx2 is required for lens marker expression until stage 25 (Steventon et al., 2012), we activated the Otx2-EnR-GR construct at stage 26 and analyzed the experiments at stage 28. While *FoxE3* expression is present in control and experimental conditions (Fig. 2Q and R, respectively), the domain appears larger upon addition of DEX. This is consistent with a role for Otx2 or its targets in controlling cell movements to form the lens, resulting in a more dispersed expression of *FoxE3*. To assess this directly, we co-injected Otx2-EnR-GR and nuclear-RFP and grafted the labelled anterior PPR into an un-injected host at stage 13. By stage 26, grafted cells are widely spread to cover much of the anterior ectoderm (Fig. 2S,T). In the absence of DEX, PPR cells in the eye region aggregate to form the lens placode by stage 30 (Fig. 2U). In contrast, when Otx2-EnR-GR is activated, cells fail to converge and do not contribute to a lens placode (Fig. 2V). Heat maps of overlaid images from eight grafted control (Fig. 2W) and experimental (Fig. 2X) embryos demonstrate the reproducibility of this phenotype.

### Otx2 is required for directional cell movements into the lens placode

**Fig. 3.**
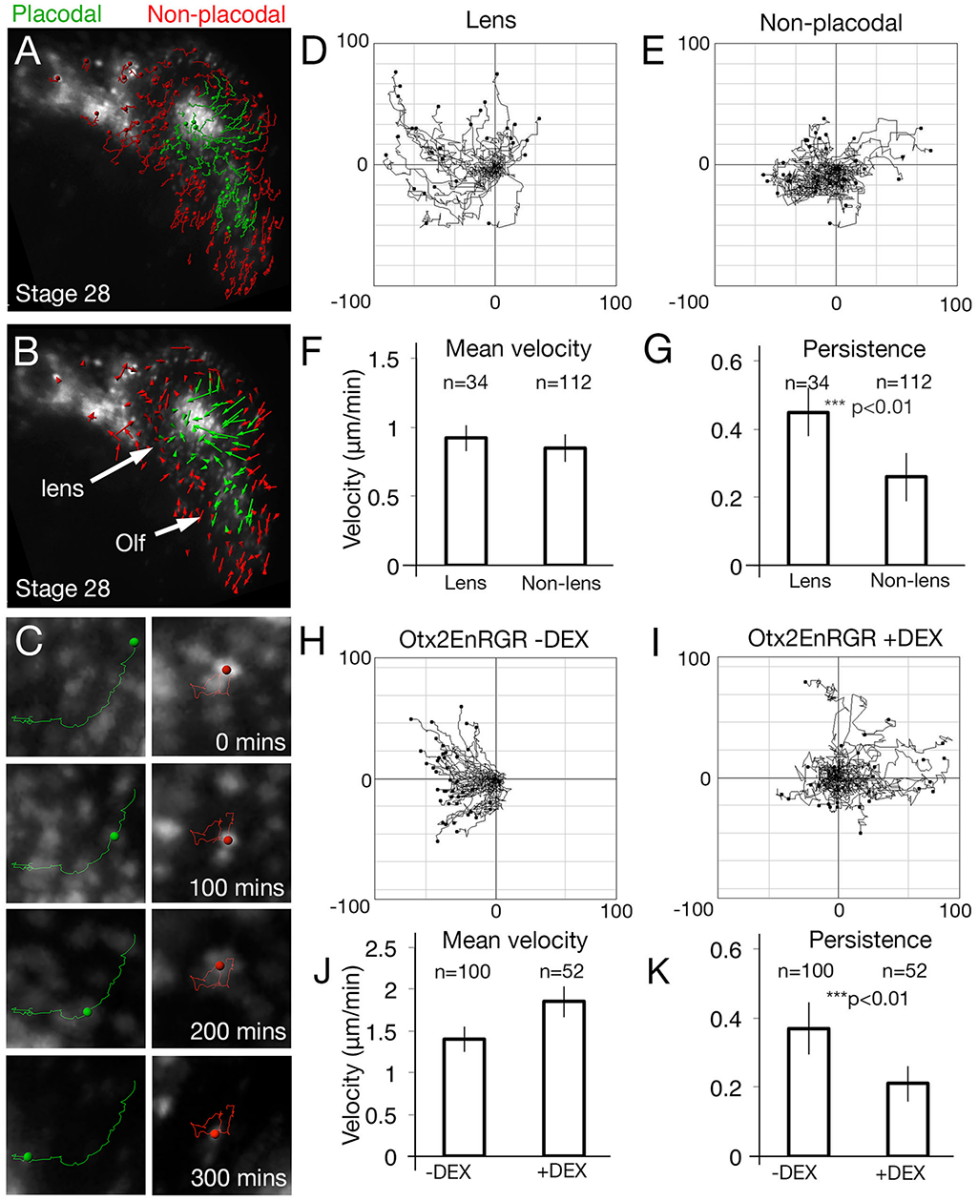
**Otx2 targets are required for directional cell movements into the lens placode.** (A,B) Automatic tracks of PPR cells sorted into placodal (green) or non-placodal (red) cells based on their final position and overlaid onto the final frame of the movie (A). (B) Cell displacements; arrows indicate the displacement of cells from their initial to their final positions. (C) Track of a single cell that enters the lens (green) or remains outside (red). (D,E) Summary diagrams of all tracked cells from Fig. 2. To compare the directionality of cells, tracks were translated so that each starts at a common origin. Displacement away from this origin is then plotted as pixel number distance from the centre point (−100 to +100 in each direction). (F,G) Mean velocity (F; *P*=0.11) and persistence (G; *P*=4.8×10^−10^) of lens and non-lens cells. (H,I) Cell tracks from PPR grafts containing Otx2-EnR-GR in the absence (H) and presence (I) of DEX. (J,K) Mean velocity (J; 1.29×10^−13^) and persistence (K; *P*=3.44×10^−11^) of cells in the absence or presence of DEX. *n*=number of cells tracked from three independent movies. Error bars:±one standard deviation either side of the mean.

To analyse whether directional cell movements accompany lens placode formation, we automatically tracked cells in the nRFP-labelled anterior placode territory (Fig. 3A,B). Tracks were colour-coded according to their final location at stage 30, either within (green) or outside (red) the placode, and filtered to include only long tracks (>10 time-points). Tracks of placode cells are longer and straighter compared to those of non-placodal cells (Fig. 3A,B). Collecting tracks from three independent movies allows statistical analysis and the assessment of persistence (the displacement distance divided by overall distance travelled) and mean velocity of cells as they move into the lens placode (Fig. 3C,D; green tracks Movie 3), or remain in the non-placodal ectoderm (Fig. 3C,E; red tracks Movie 3). Both cell populations move with the same velocity (Fig. 3F), however, the persistence of lens cells is significantly increased when compared to non-lens cells (Fig. 3G), and we confirmed this by manually tracking a subset of lens and non-placodal cells (persistence: *P*=1.7×10^−5^, velocity: *P*=0.075, non-lens=37, lens=29). Taken together, these results demonstrate that although all cells in the PPR move with the same mean speed, lens cells move with increased persistence.

To determine how Otx2 and its targets affect lens formation, we analysed cell behaviour in embryos with nRFP-labelled PPR grafts from Otx2-EnR-GR injected embryos. Automated cell tracks were generated from embryos cultured in absence (Fig. 3H, *n*=100 cells) or presence of DEX (Fig. 3I, *n*=52 cells). As no morphological placode forms in this case, we were unable to analyse placodal and non-placodal tracks separately. Despite this, repression of Otx2 targets led to an overall decrease in persistence (Fig. 3K). We observed a minor increase in speed compared to controls, although this was not significant (Fig. 3J). These results were confirmed by the manual tracking of a subset of cells (*P*=1.31×10^−11^, n: −DEX= 32, +DEX=35). Thus, during placode condensation, activation of Otx2 targets is required for persistent cell movements and for cells to integrate into the forming lens.

### Gbx2 targets are required for directed movement of cells into the otic placode

**Fig. 4.**
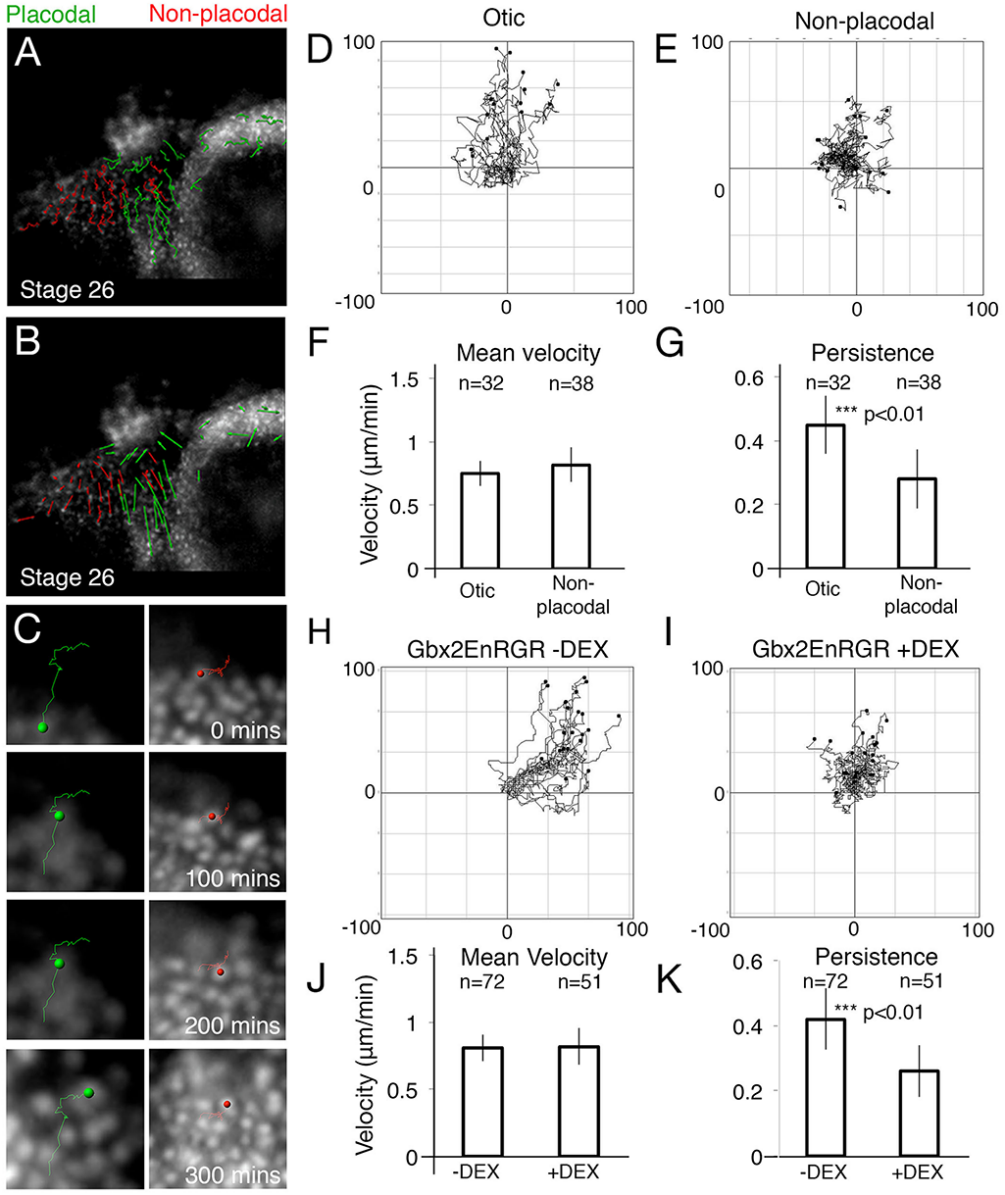
**Gbx2 targets are required for directional cell movements into the otic placode.** (A,B) Automatic tracks of PPR cells sorted into placodal (green) or non-placodal (red) cells based on final position and overlaid onto the final frame of the movie (A). (B) Cell displacements, arrows indicate the displacement of cells from their initial to final positions. (C) An example of five cells that were automatically tracked in a region posterior and ventral to the forming otic placode. (D,E) Summary diagrams of all tracked cells. To compare the directionality of cells, tracks were translated as in Fig. 3. (F,G) Student *t*-tests (2-tailed) of otic, epibranchial (epi) and non-placodal cell movements to analyse mean velocity (F; *P*=0.18) and persistence (G; *P*=2.5×10^−4^). (H,I) Cells were tracked from PPR grafts containing Gbx2-EnR-GR in the absence (H) and presence (I) of DEX from stage 18. (J,K) Students *t*-tests (2-tailed) of cells carrying Gbx2-EnR-GR in the presence or absence of DEX to analyse mean velocity (J; *P*=0.51) and persistence (K; *P*=1.5×10^−3^) of cell movements. *n*=number of cells tracked from three independent movies. Error bars: ±one standard deviation either side of the mean.

Do similar directional movements occur as the posterior placode territory divides into discrete placodes? nRFP-labelled posterior PPRs were grafted into unlabelled host embryos of the same stage to follow placode cell movements in the posterior PPR. Cell tracking reveals that otic or epibranchial progenitors (Fig. 4A,B; green, Movie 4) display an increase in both track length (Fig. 4A) and overall displacement (Fig. 4B) when compared epidermal cells (Fig. 4A,B; red, Movie 4). Although there is no significant difference in the average speed (Fig. 4F) of otic (Fig. 4D) and non-otic cells (Fig. 4E), otic cells move with increased persistence (Fig. 4G). Manual tracking confirms these results (persistence: *P*=3.8×10^−4^, velocity: *P*=0.014, n: otic=14, non-placodal=17).

We next assessed the role of Gbx2 and its targets in directional migration of cells into the otic placode. Automatic cell tracking reveals that when Gbx2 targets are repressed, the persistence of movement is decreased (Fig. 4I,K *n*=51 cells) as compared to controls (Fig. 4H,K *n*=72 cells), although no significant change in mean speed is observed (Fig. 4J). Manual tracking of cells confirms this decrease in persistence (persistence: *P*=1.09×10^−10^, velocity: *P*=0.141, n: −DEX=21, +DEX=19). Taken together, these results demonstrate that Gbx2 has a role in controlling directional cell movements in the otic placode.

In conclusion, Gbx2 and Otx2 play multiple roles during placode formation. At neurula stages they subdivide the PPR at the otic/trigeminal boundary and are required for cells to adopt specific placode fates (Steventon et al., 2012). Later, their targets are involved in controlling directional cell movements during the formation of distinct placodes (this study).

The current study made use of inducible constructs to assess the role of Gbx2 and Otx2 at late placode stages. Unlike at placode progenitor stage (Steventon et al., 2012), these factors are no longer required for the expression of placode-specific markers at the later stages examined in this study. After activation of Gbx2-EnR-GR and Otx2-EnR-GR, placode markers are expressed in approximately the correct location, however, cells are more wide spread and placodes have abnormal shapes suggesting that Gbx2 and Otx2 control cell behaviour. Whether the endogenous proteins act as transcriptional repressors or activators is currently unknown and will require the identification of their targets in the future.

Cell movements have previously been shown to accompany placode formation in different species (Bhat and Riley, 2011; Kwan et al., 2011; Streit, 2002; Theveneau et al., 2013). A previous study in *Xenopus* showed that limited directional movements are observed within the pre-placodal region at mid-to-late neurula stages (Pieper et al., 2011). Here we show that at later stages in *Xenopus* directional movements do indeed accompany the formation of morphologically distinct placodes. We demonstrate for the first time that unlike future epidermal cells placode progenitors move directionally and that this behaviour is important for the assembly of placodes with normal morphology.

## MATERIALS AND METHODS

### Embryo techniques

*Xenopus* embryos were obtained as described previously (Steventon et al., 2012) and staged according to Nieuwkoop and Faber (1967). Plasmids were linearized; RNA transcribed using SP6 or T7 RNA polymerases, and the GTP cap analogue (Harland and Weintraub, 1985). To repress Gbx2 and Otx2 downstream targets, their homeodomain was fused to the repressor domain of engrailed and the hormone-inducible GR domain (Otx2-EnR-GR and Gbx2-EnR-GR; Glavic et al., 2002). All mRNAs were mixed with diethylpyrocarbonate (DEPC)-treated water to a concentration of 500 pg/μl, with the exception of nuclear RFP, which was used at 200 pg/μl. The authors confirm that all experiments within this article conform to the relevant regulatory standards of the UK.

### Image analysis

Embryos were prepared for live imaging as described (Theveneau et al., 2013). Automatic cell tracks were generated with Imaris (Bitplane). Manual tracking was performed using the MTrack2 plug-in for ImageJ (http://fiji.sc/). Tracks were exported and analysed for velocity and persistence of movement. All *P*-values were derived from a two-tailed Students *t*-test. To overlay images of placode grafts, images were cropped to the same size with reference to the dorsal edge of the neural tube and retina to ensure the comparison of equivalent regions. Images were overlaid in Image J, and displayed with the Fire LUT tool to display mean intensity across multiple samples. Surface rendering of images was created using the ‘surface function’ in Imaris. In order to highlight coherent regions of labelled cells, the automatically segmented regions were colour-coded by area, with a shift from yellow to red as the area decreased.

### *In situ* hybridisation

*Xenopus* embryos were prepared, hybridized and stained as previously described (Harland, 1991), and NBT/BCIP or BCIP alone were used to reveal the signal. The genes analyzed were *Eya1* (David et al., 2001), *Pax2* (Heller and Brandli, 1999), and *FoxE3* (Kenyon et al., 1999).
